# Simultaneously Achieved High Piezoelectricity and High Resistivity in Na_0.5_Bi_4.5_Ti_4_O_15_-Based Ceramics with High Curie Temperature

**DOI:** 10.3390/ma17235857

**Published:** 2024-11-29

**Authors:** Zhengli Huan, Ning Chang, Yunyun Feng, Xuan Fei, Xiang Xu, Huiming Ji

**Affiliations:** 1 Key Laboratory of Advanced Ceramics and Machining Technology of Ministry of Education, School of Materials Science and Engineering, Tianjin University, Tianjin 300350, China; huanzhengli@sina.com; 2 Shandong Liensi Intelligent Technology Co., Ltd., Dezhou 253000, China; youxiangfyy@163.com (Y.F.); q1427026256@163.com (X.F.); xuxiang971218@163.com (X.X.)

**Keywords:** Na_0.5_Bi_4.5_Ti_4_O_15_, Na_0.5_Bi_3_(LiMn)_0.9_Ti_4_O_15_, high-temperature piezoceramics, resistivity

## Abstract

Good piezoelectricity and high resistivity are prerequisites for high-temperature acceleration sensors to function correctly in high-temperature environments. Bismuth layered structure ferroelectrics (BLSFs) are promising candidates for piezoelectric ceramics with excellent piezoelectric performance at high temperatures, high electrical resistivity, and high Curie temperatures (*T*_c_). In this study, (LiMn)^5+^ is substituted for Bi at the A-site, and Ce-doping is performed to replace Ti ions in Na_0.5_Bi_4.5_Ti_4_O_15_, which achieves the desired combination of high piezoelectric coefficients and high resistivity. Herein, we prepared Na_0.5_Bi_3_(LiMn)_0.9_Ti_4−x_Ce_x_O_15_ high-temperature piezoelectric ceramics, achieving a high piezoelectric coefficient *d*_33_ of 32.0 pC/N and a high resistivity ρ of 1.2 × 10^8^ Ω·cm (at 500 °C), and a high Curie temperature of 648 °C. It is important that the *d*_33_ variation remains within 8% over a wide temperature range from 25 °C to 600 °C, demonstrating excellent thermal stability. Structural characterization and microstructure analysis showed that the excellent piezoelectric coefficient and high resistivity of cerium-doped Na_0.5_Bi_4.5_Ti_4_O_15_-based ceramics are attributable to the synergistic effects of structural characteristics, defect concentration, refined grain size and domain morphology. This study demonstrates that the superior properties of Na_0.5_Bi_3_(LiMn)_0.9_Ti_4−x_Ce_x_O_15_ ceramics are crucial for the stable operation of high-temperature accelerometer sensors and for the development of high-temperature devices.

## 1. Introduction

Piezoelectric materials belong to a group of materials capable of converting electrical energy into mechanical energy and vice versa. Owing to their unique characteristics, these materials have found widespread applications in military weapons, artificial intelligence, aerospace, electronics, medical devices, biology and energy sectors [1,2,3,4]. Recent advances in sensor technology, particularly in automotive engine monitoring, nuclear energy monitoring, and aircraft engine monitoring, require sensors that can operate stable in high-temperature environments [5,6]. Consequently, high-temperature piezoelectric materials with exceptional piezoelectric properties are becoming increasingly important.

Bismuth layered-structure compounds (BLSFs) were first discovered by Aurivillius et al. in 1949 [7]. BLSFs are layered structure oxides with a unique crystal structure and high Curie temperature (*T*c). The general formula is (Bi_2_O_2_)^2+^(A_m−1_B_m_O_3m+1_)^2−^, which consists of alternating (Bi_2_O_2_)^2+^ and pseudo-perovskite layers (A_m−1_B_m_O_3m+1_)^2−^ arranged along the c-axis in a specific pattern. A is a +1 to +4 valence cation or a combination suitable for 12-coordination, such as Na^+^, Ba^2+^, Bi^3+^, U^4+^, etc. B is a +3 to +5 valence small-radius cation suitable for 6-coordination or a combination thereof, such as Cr^3+^, Ti^4+^, Nb^5+^, W^6+^, etc. m is the number of layers in the pseudo-perovskite layer, usually a positive integer from 1 to 5 [8,9,10,11,12,13]. Examples include Bi_4_Ti_3_O_12_ (m = 3), SrBi_4_Ti_4_O_15_ (m = 4), SrBi_4_TiO_18_ (m = 5) and others. Bismuth-layered oxide ceramics have high Curie temperature, making them promising for high-temperature, harsh environment applications. Bismuth layered oxides such as Bi_4_Ti_3_O_12_, CaBiNb_2_O_9_, CaBiTi_4_O_15_, BiTiNb_3_O_9_, etc., are considered excellent candidates for aerospace, biomedicine, geological surveys, military applications and high-temperature sensors due to their high *T*_C_ values and high electromechanical coupling coefficients. More studies focus on BLSFs with superior comprehensive performance to meet the demands of high-temperature piezoelectric devices. For instance, the stabilizing piezoelectric constant *d*_33_ affects the sensor’s sensitivity, while high resistivity helps prevent sensor medium breakdown. However, achieving ceramics that exhibit higher *T*_C_ values, larger *d*_33_ and improved resistivity simultaneously remains challenging. Previous studies demonstrated that oxygen vacancy defects, resulting from the unavoidable volatilization of bismuth in BLSFs, degrade the piezoelectric properties and resistivity of the ceramics [14,15]. Consequently, commonly used strategies include the low sintering temperature (*Ts*) and substituting elemental Bi with other ions to reduce the concentration of oxygen vacancies. Additionally, doping modifications can considerably enhance the piezoelectric properties of BLSF ceramics, particularly when substituting A or B-site ions, which can change the crystal structure and spontaneous polarization (*Ps*) behavior. For instance, Li et al. [16] replaced the B-site ions in Bi_4_Ti_3_O_12_ with a combination of high-valent Ta^5+^ and low-valent Cu^2+^, resulting in high piezoelectric constants (~27 pC/N). Similarly, Sheng et al. [17] doped CaBiTi_4_O_15_ with Nd^5+^ and Ce^4+^, achieving not only high piezoelectric constants (~19 pC/N) and high *T_C_* (~794 °C) but also optimizing the overall properties. Reports indicate that excessive doping with Bi and Ce can effectively increase the samples’ resistivity. For example, Chen et al. [18] achieved a high resistivity ρ_500 °C_ = 3.7 × 10^9^ Ω·cm by cooping Ce and W into CaBi_2_Nb_2_O_9_ ceramics. Li et al. obtained a resistivity of 2 × 10 Ω·cm at 600 °C by increasing the Bi ion content in the (Bi_2_O_2_)^2+^ layer in the CaBi_2_Nb_2_O_9_ matrix. However, simultaneously achieving both high piezoelectric constants and high resistivity in piezoelectric ceramics remains challenging.

Na_0.5_Bi_4.5_Ti_4_O_15_ (NBT) ceramics feature a structure with two bismuth layers interspersed with four chalcogenide layers. These ceramics are notable for their high Curie temperature (*T*c = 655 °C), low dielectric losses and low *Ts*, making them suitable for high-temperature applications while reducing energy consumption during production [19]. To achieve piezoelectric ceramics with high piezoelectric constants and high resistivity with enhanced thermal stability, we selected Na_0.5_Bi_4.5_Ti_4_O_15_ matrix ceramics for this study. For this study, Ce-doping to replace Ti ions in Na_0.5_Bi_4.5_Ti_4_O_15_ with Li/Mn in substitution for Bi at the A-site is proposed to boost the piezoelectric performance of NBT-based piezoceramics, including piezoelectric constant and resistivity. 

## 2. Materials and Methods

Na_0.5_Bi_3_(LiMn)_0.9_Ti_4−*x*_Ce*_x_*O_15_ (NBLMTC-*x*, *x* = 0.0, 0.2, 0.4, 0.6, and 0.8) ceramics were prepared using a solid-phase synthesis method. Raw materials included Na_2_CO_3_ (99.8%), Bi_2_O_3_ (99.99%), Li_2_CO_3_ (99.9%), MnO_2_ (91.8%), CeO_2_ (99.99%) and TiO_2_ (99.5%). The powders were weighed according to the stoichiometric ratios, mixed with ethanol as the milling medium and ball-milled for 8 h. The mixture was then dried and calcined at 850 °C for 3 h. The calcined powder was ball-milled again, dried and pelletized by adding 9 wt% polyvinyl alcohol (PVA) binder. The granules were shaped into columns using powder tablet presses. Then, PVA was removed by heating at 600 °C, and the pellets were sintered at various temperatures for 3 h.

The crystal structure of the NBLMTC-x samples was analyzed using an X-ray diffractometer (D8, ADVANCE; Bruker AXS GMBH, Karlsruhe, Germany). Raman spectra in the 200~1000 cm^−1^ range were tested at room temperature with a Lab RAM HR800 instrument (LabRAM HR, HORIBA, Longjumeau, France). Dielectric properties and complex impedance properties of all samples at 10 kHz were obtained using a precision LCR impedance analyzer (HP4294A, Agilent, Santa Clara, CA, USA) in the temperature range of 25–700 °C. Additionally, the *ρ*_dc_ resistivity of all samples was tested using a high resistivity meter (DMS 1000, Bailibo, Wuhan, China) in the temperature range of 50 °C~600 °C. The ferroelectric properties were characterized using a ferroelectric analyzer (FEAI1000, Bailibo, Wuhan, China). All samples were polarised in silicone oil (MPT 50 KVOP, Bailibo, Wuhan, China) at 180 °C for 30 min under an applied DC field of 10~12 kV/mm. Subsequently, their piezoelectric properties were evaluated using a piezoelectric-d_33_ tester (ZJ-3A, Institute of Acoustics, Beijing, China).

## 3. Results

**Figure 1:**
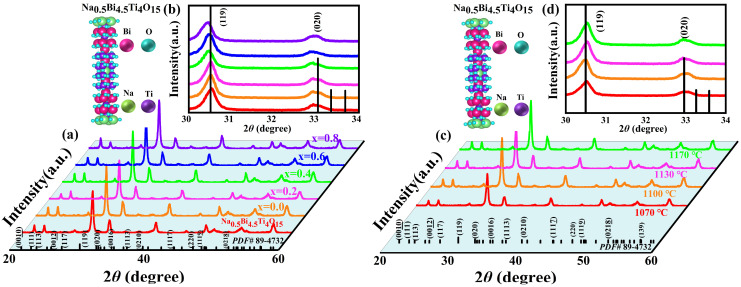
(**a**) XRD patterns of NBLMTC-*x* ceramics at room temperature. (**b**) (119) Local magnification of the peak. (**c**) XRD patterns of *x* = 0.4 ceramic samples sintered at various temperatures. (**d**) (119) Local magnification of the peak.

Figure 1 shows the XRD patterns of NBLMTC-x ceramics with various compositions and Ts values. Figure 1a shows the X-ray diffraction (XRD) patterns of NBLMTC-x piezoelectric ceramics sintered at 1100 °C. All ceramics exhibit a layered perovskite oxide structure (NBT, PDF 89-4732) with no secondary phase impurities. The principal diffraction peaks of all samples align with the main phase of NBT ceramics, indicating the successful incorporation of Ce into the NBT attice. Figure 1b depicts a magnified view of the XRD patterns near 30° and 34° from Figure 1a. The (119) diffraction peak shifts slightly to a lower angle, reflecting lattice expansion with increasing Ce concentration. Depending on the tolerance factor, a dopant element will replace the B-site if its radius r is less than 87 pm and the A-site if r is greater than 94 pm. If the radius falls between these two ranges, the dopant may replace either the A or B sites. Ce^4+^ (12-coordination) with a radius of 87 pm can replace Ti^4+^ (12-coordination) [20] at the B-site. Because Ti^4+^ has a smaller radius of 56 pm compared to Ce^4+^, substituting Ti^4+^ with Ce^4+^ increases the lattice constant, causing the diffraction angle of the sample to shift to a lower angle. It is important to note that the (119) peak of the Na_0.5_Bi_4.5_Ti_4_O_15_ sample shifts to a slightly higher angle. This shift is attributable to the formation of oxygen vacancies caused by the volatilization of bismuth at high temperatures, leading to the contraction of the crystal lattice and a reduction in crystal plane spacing. Compared to the peak shift direction for the sample with x = 0, it can be inferred that substituting Bi ions with (LiMn)^5+^ at the A-site in NBT may inhibit elemental Bi volatilization. Figure 1c shows the XRD patterns of NBLMTC-x ceramics sintered at various temperatures for *x* = 0.4. All samples correspond to the primary NBT phase, with no secondary phases detected. Figure 1d depicts a magnified view of the (119) peak from Figure 1c. It shows that while the diffraction angle of the main (119) peak shifts to a lower angle initially, it subsequently shifts to a higher angle as the temperature increases. This high-temperature shift is attributed to the formation of oxygen vacancies from Bi volatilization, causing the crystal lattice to contract, decreasing the crystal plane spacing and resulting in a shift of the XRD peaks to a higher angle. Therefore, combined with the observations in Figure 1b,d, it can be seen that some Ce^4+^ ions replace Ti^4+^ in the NBT lattice.

**Figure 2:**
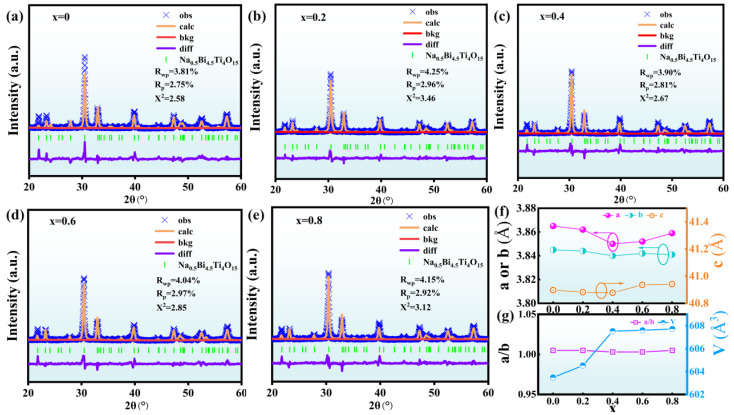
(**a**–**e**) Rietveld refinement of XRD patterns for NBLMTC-*x* ceramics. (**f**,**g**) Variation of lattice constants a, b, c and cell volume V as a function of *x*.

XRD refined diffraction patterns were analyzed using General Structure Analysis Software (GSAS-II) to assess the impact of Ce doping on the structure of NBLMTC-x ceramics and to extract the lattice constants a, b and c, as well as the cell volume V. Figure 2a–e show the refined XRD patterns for NBLMTC-x samples (x = 0.00, 0.2, 0.4, 0.6 and 0.8), which closely match the original XRD patterns and exhibit low R_wp_, R_p_ and χ^2^ values (R_wp_ ≤ 5%, R_p_ ≤ 4% and χ^2^ ≤ 3.5%). These values strongly agree with the refined computational data and the experimental results. As shown in Figure 2f, the lattice constants a, b and c of the NBLMTC-x ceramics decrease at *x* = 0.4. However, as the doping amount increases, the lattice constants a, b and c gradually increase. The degree of orthorhombic distortion in the ceramic samples can be characterized by the quadratic factor a/b [21,22,23]. Figure 2g illustrates that the a/b value slightly decreases with the addition of Ce, and the tetragonality factor a/b of the doped samples approaches 1. This result indicates that Ce doping can reduce the anisotropy of the a–b plane in the NBT ceramics. Additionally, the gradual increase in the cell volume V is consistent with the previously observed shift in the (119) peak from the XRD data. As shown in Figure 2g, this increase can be attributed to the replacement of smaller Ti^4+^ (0.56 Å) with the larger Ce^4+^ ions (0.87 Å), enlarging the cell volume.

**Figure 3:**
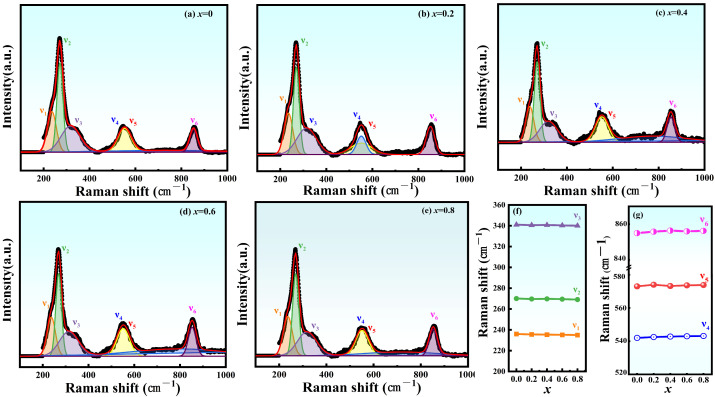
(**a**–**e**) Raman spectra of NBLMTC-*x* ceramics (*x* = 0.00–0.8). (**f**,**g**) Variation in peak shifts of each mode with respect to *x*.

Raman spectra were collected to explore the structural properties of the samples further. Figure 3a–e depicts the Raman spectra of NBLMTC-x piezoelectric ceramics measured at room temperature in the 200–1000 cm^−1^ range. The spectra of all samples were fitted using the Lorentzian function. The peak positions of the various modes aligned with those observed in previous studies of NBT ceramics. The fitted spectra reveal six major bands, labeled as ν_1_–ν_6_, at approximately 238, 272, 343, 560, 580 and 855 cm^−1^. In the lower frequency range, the vibrational spectra are related to the interactions between the cation–octahedron and the surrounding groups, originating from both the internal and external vibrations of the Ti–O octahedron [24]. The Ti–O octahedra’s internal stretching and bending vibrations appear in the spectral region above 200 cm^−1^ [24]. The strongest vibrational mode, ν_2_, represents the strongest bending vibration of the TiO_6_ octahedron, which is attributed to its F_2g_ symmetry property. In contrast, the vibrational modes ν_1_ and ν_3_ are activated by the twisting of the Ti–O octahedron, attributed to the F_2μ_ and F_1μ_ symmetries, respectively [25]. As shown in Figure 3a, the broad peak around 560 cm^−1^ is associated with the symmetry, while the two characteristic components of the split orthogonal structure, ν_4_ and ν_5_, exhibit a gradual merging with increasing Ce doping. This merging suggests Ce doping reduces the degree of orthogonal distortion, aligning with the XRD refinement results. The ν_6_ mode corresponds to another bond-stretching vibration with A1g symmetry [26]. The gradual shift of the ν_6_ mode toward the high-frequency band suggests Ce substitution occurs at the B site in the BNT, as shown in Figure 3f–g. Based on the Raman results and XRD refinement analysis, it is evident that Ce doping replaces Ti atoms at the B-site in NBT. This substitution affects the distortion of the Ti–O octahedron, thereby reducing the degree of orthorhombic distortion and leading to considerable changes in *Ps*.

**Figure 4:**
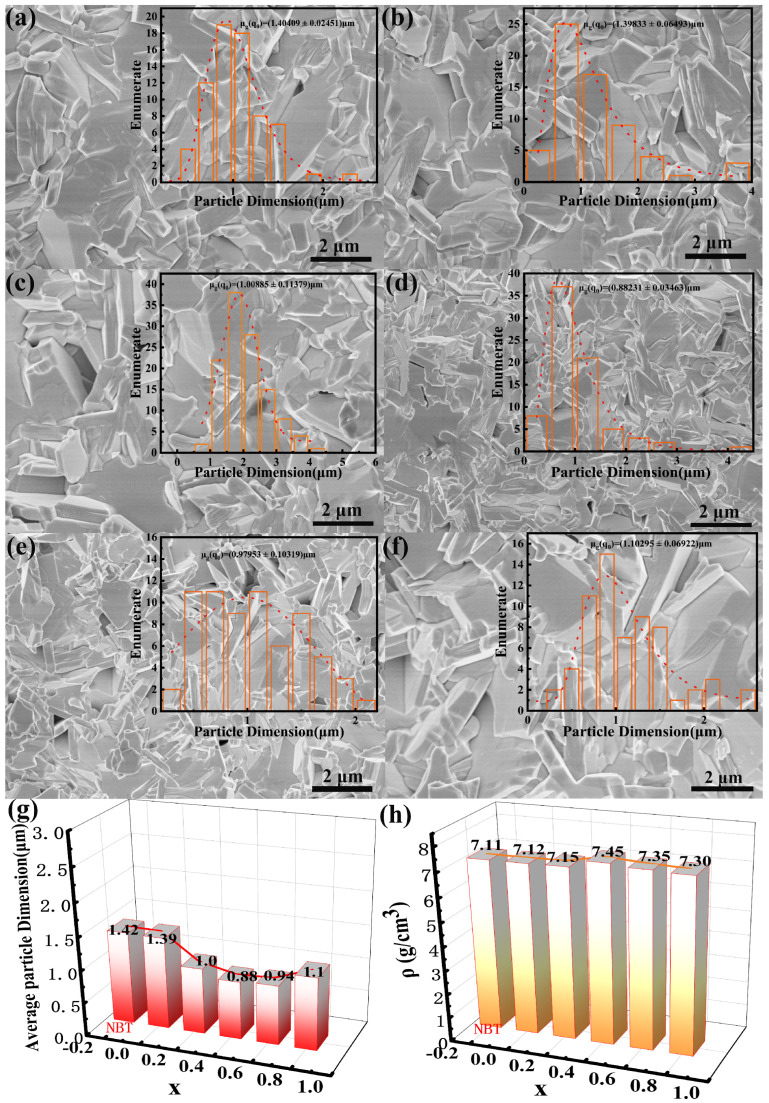
(**a**–**f**) SEM images of the cross-section NBLMTC-*x* ceramics; (**g**) The average grain size of the NBLMTC-*x* ceramics. (**h**) The measured density of the NBLMTC-*x* ceramics.

Figure 4a–f presents cross-sectional SEM images of NBT and NBLMTC-x ceramics. The plate-like grains observed in all samples are consistent with the distinctive characteristics of BLSF ceramics [27,28]. As shown in the insets of Figure 4a–f, the NBT sample has the largest grain size, nearly identical to that of the NBLMTC-x sample with x = 0. The grain size of the NBLMTC-x ceramics initially decreases and then increases with increasing x. The grain boundaries are clearly defined and densely packed, indicating that all samples have minimal defects. This result reduces leakage currents in the NBLMTC-x ceramics at high temperatures. Grain size and grain boundary resistance are inversely related, with the resistance at grain boundaries generally higher than that in the grains. This increased resistance is due to the disordered atomic arrangement near the grain boundaries, which enhances electron scattering and increases resistivity. The sample grain sizes were measured using Nano Measure software 1.2.5, as shown in Figure 4g. It is evident that the grain size trends decrease and then increase with Ce doping, with the smallest grains observed in the samples with x = 0.4, with an average size of 0.80–0.88 μm. As shown in Figure 4f, the sample density was also measured, revealing a trend of increasing and decreasing, with a peak density of approximately 7.45 g/cm^3^ at x = 0.4. This trend is opposite to that of the grain size changes, suggesting that Ce doping effectively reduces the grain size of the samples. The tightly packed grain boundaries enhance the densification of the ceramics, thereby increasing their density. These factors contribute to the ceramics’ ability to maintain low leakage currents at high temperatures, improving their high-temperature resistors.

**Figure 5:**
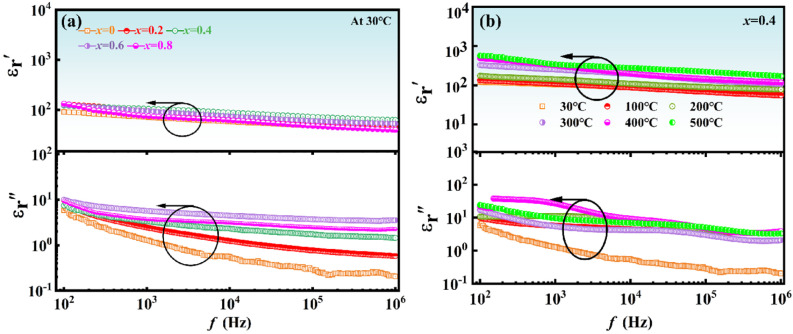
Frequency evolution of the real and imaginary part of relative dielectric constant (log-log scale) of NBTLM-*x* ceramics: (**a**) x = 0, 0.2, 0.4, 0.6, 0.8. (**b**) The presentation of detected complex dielectric permittivity spectra for the tests NBTLM-x ceramics (*x* = 0.4) in the tested temperature range.

The frequency evolution for the real and imaginary parts of the relative dielectric constant (log-log scale) of NBTLM-x ceramics is depicted in Figure 5. The dielectric properties of piezoelectric materials are characterized by the relative dielectric constant ε_r_, which can be expressed using the Formulas (1) and (2):

(1) $$\varepsilon =\frac{Cd}{\varepsilon_{0}A}$$

(2) $$\varepsilon_{r}=\frac{\varepsilon }{\varepsilon_{0}}=\frac{t\times C}{S\times \varepsilon_{0}}$$

where: ε is the dielectric constant; C is the capacitance, F; d is the thickness, m; *ε*_0_ is the relative dielectric constant of the ceramic sample; A is the area of the sample, m^2^; *ε*_0_ is the relative dielectric constant of the ceramic sample, which has a value of about 8.85 × 10^−12^ F/m.

From Figure 5a, the static domain manifested via “horizontal changes” in scan within the frequency range 100 Hz < *f* < 1 MHz is the common feature and frequency offset does not significantly change the dielectric constant value of NBTLM-*x* ceramics, which is related to its relationship with the so-called static spectral domain. This is also the definition of the canonical dielectric constant [29]. The dielectric constant is the basic property whose temperature evolution is considered for relaxor ceramics. The presentation of detected complex dielectric permittivity spectra for the tests NBTLM-*x* ceramics (*x* = 0.4) in the tested temperature range indicated in Figure 5b. It can be seen that the frequency shift has a negligible effect on the measured values. This result is consistent with the canonical definition of the dielectric constant in Dielectrics Physics [30,31], which defines it as the nearly constant value of ε′ = ε in the static frequency domain. 

**Figure 6:**
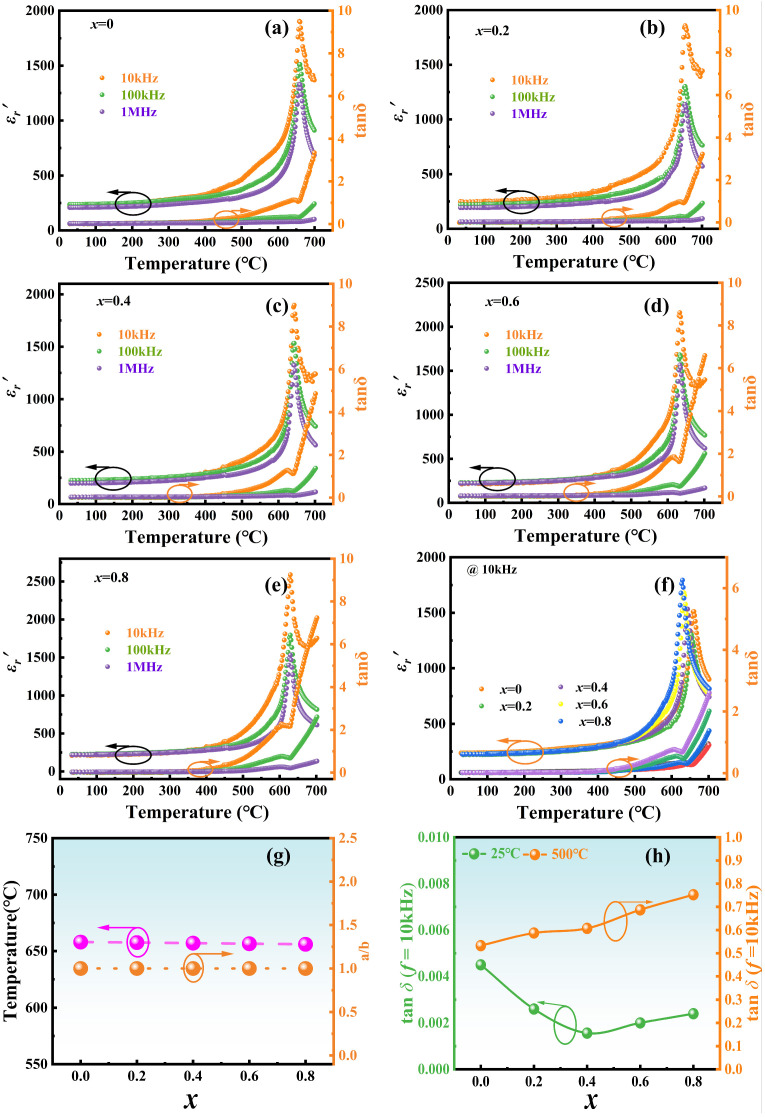
(**a**–**e**) The real part of the relative dielectric constant of NBTLM-x ceramics measured at different frequencies and the change of dielectric loss with temperature. (**f**) Variation of the dielectric constant properties of NBTLM-*x* ceramics as a function of temperature, including the real part of the dielectric constant and the dielectric loss, tested at selected frequencies. (**g**) The Curie temperature (*T*c) and the a/b value as a function of x = 0.00–0.8. (**h**) variation of dielectric loss with composition at 25 °C and 500 °C.

Figure 6a–e presents the temperature dependence of dielectric properties, including the relative dielectric constant real part and dielectric loss tanδ, of NBLMTC-x ceramics measured at different frequencies. From Figure 6a–e, all of the samples have the same trend at various frequencies, and the trend of their real part of the relative dielectric constant is comparatively flat below 500 °C, which shows that the ceramic samples exhibit good thermal stability. After the temperature exceeds 500 °C, the real part of the relative dielectric constant of the sample starts to rise sharply and decreases rapidly after reaching a maximum value of about 650 °C. In addition, the same sample shows a decreasing trend in its dielectric constant with increasing frequency. This is due to the presence of many defective dipoles and ionic conductivity within the material; whereas defective dipoles require a long relaxation time for directional migration within the material, space charge polarization responds more quickly at lower frequencies and is more temperature dependent. At higher frequencies, the directional migration of the defective dipole cannot keep up with the change in the electric field and exhibits a hysteresis phenomenon, and the dipole does not contribute to the real part of the relative permittivity, resulting in a lower real part of the material’s phase permittivity at high frequencies. The variation in dielectric loss with temperature at different frequencies is consistent with the real part of the relative dielectric constant, which remains nearly unaltered from room temperature to 500 °C. At temperatures above 500 °C, the dielectric loss tanδ gradually increases. When the temperature approaches *T*_C_, the rate of increase accelerates significantly. This is due to the ceramics being affected by leakage conduction at high temperatures, causing the loss to increase sharply. Meanwhile, the identical ceramic sample displays a considerable drop in dielectric loss as frequency increases.

To compare the effect of doping x on Curie temperature, the real part of the relative dielectric constant, and dielectric loss of piezoelectric ceramics. Figure 6f shows the temperature dependence of dielectric properties, including the relative dielectric constant real part and dielectric loss tanδ of NBLMTC-x ceramics measured at 10 kHz. Figure 6f demonstrates that the actual part of the relative dielectric constant steadily increases with temperature in all NBLMTC-x ceramics but drops after reaching the phase transition temperature (*T*_m_) from ferroelectric to paraelectric. The transition from the ferroelectric phase to the cis-phase results in a consistent dielectric peak around 600~660 °C across all samples, showing that their *T*_C_ values fall within this temperature range. Unlike usual ferroelectrics, the temperature dependence of the dielectric constant of a relaxing ferroelectric does not conform to the Curie-Weiss law but to a modified Uchino-Nomura equation [32,33]:

(3) 1εr′−1εm=T−TmγC

In this equation, ε_m_ is the maximum value of the dielectric constant; $\varepsilon_{r}^{\prime }$ denotes the real part of the dielectric constant at T temperature; *T*_m_ represents the temperature at which the dielectric constant peaks; C is the Curie constant; and γ is the dispersion factor, which is used to describe the degree of dispersion of the phase transition. Usually, γ = 1 denotes that the material is a normal ferroelectric; 1 < γ < 2 denotes that the material belongs to the relaxation ferroelectric; γ = 2 indicates that the material is an ideal relaxation ferroelectric.

Dielectric loss results from the dielectric-containing carriers that can conduct electricity; these carriers, in the function of the applied electric field, produce conductive current, thereby consuming part of the electrical energy and transforming it into a thermal energy phenomenon. As can be seen in Figure 6f, the tanδ of different NBLMTC-*x* ceramics does not change significantly before 500 °C. When the temperature reaches 500 °C, the tanδ grows rapidly. This phenomenon is caused by carriers being free to travel in higher temperature conditions, such as thermally induced space charge transfer. DC conductivity takes over the entire dielectric reaction process.

The magnitude of the *T*_C_ is closely related to the lattice distortion, with large lattice distortions corresponding to high *T*_C_. The lattice distortion can be reflected by the a/b value as aforementioned. Ce-doping causes tanδ to increase slowly with increasing temperature at 25 °C~500 °C (Figure 6g). When the temperature is higher than 500 °C, the tanδ of the NBLMTC-*x* samples escalates substantially, likely owing to the activation of carriers that enhance conductivity and exacerbate losses at high temperatures. Figure 6h summarises the tanδ values of NBLMTC-*x* ceramics at 25 °C and 500 °C. At 25 °C, tan δ remains low (<0.2%), and it is still below 0.8% at 500 °C. Therefore, Ce-doped samples exhibit promising potential for use in high-temperature applications.

**Figure 7:**
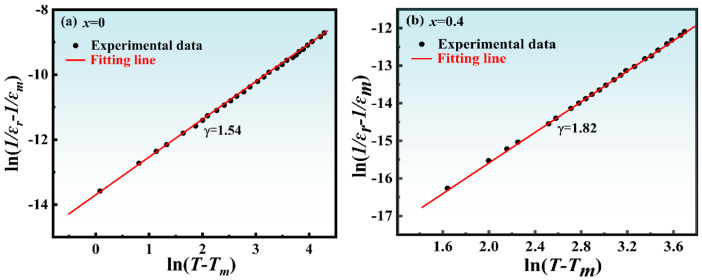
(**a**,**b**) Plots ln(*1/ε_r_* − *1/ε_m_*) vs. ln(*T* − *T*_m_) for NBTLM-x ceramics above *T*_m_ (10 kHz).

To validate the ferroelectric type of NBLMTC-x. Using Equation (3), fitting the variation of ln(1/*ε_r_* − 1/*ε_m_*) versus ln(*T* − *T_m_*) for NBLMTC-x ceramics at 10 kHz yields the relaxation degree of the ceramics, γ. Figure 7 shows the NBLMTC-x ceramic ln(1/*ε_r_* − 1/*ε_m_*) plotted against ln(*T* − *T_m_*) at 10 kHz. It is discovered that the sample’s γ is 1.54 at x = 0 and rises to 1.82 at x = 0.4. The primary cause of the change in relaxation degree is Ce^4+^ replacing Ti^4+^ in the crystal lattice, which increases the disorder of the B-site cation arrangement and causes the ceramic composition to be inhomogeneous. Additionally, the crystal contains numerous micro-zones with varying phase transition temperatures, which help to extend the phase transition temperature of the cis-phase and ferroelectric phase into The γ-value of the Ce^4+^ doped ceramic sample is about 2, indicating that the doped sample is very close to the optimal relaxation ferroelectric. The Curie temperature zone gives the ceramics diffuse phase transition characteristics.

**Figure 8:**
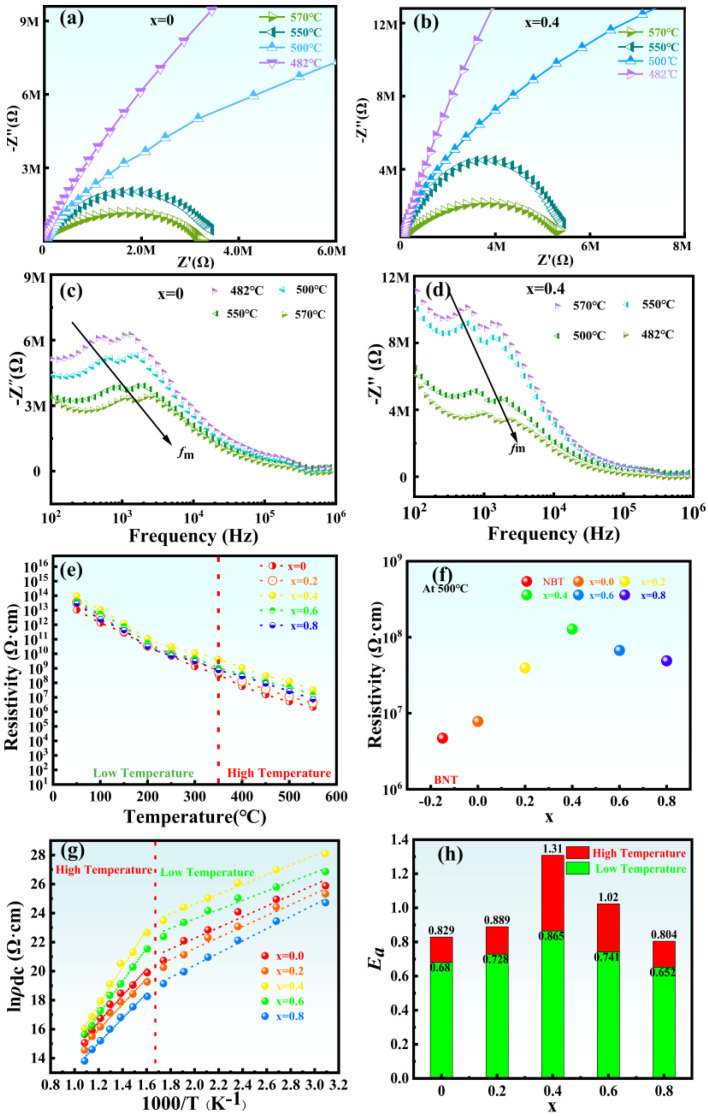
(**a**,**b**) Cole-Cole curves of x = 0 and x = 0.4 ceramic samples at different temperatures. (**c**,**d**) Frequency dependence of the imaginary part (Z″) of the complex impedance of NBLMTC-*x* ceramics at different temperatures. (**e**) The temperature dependence of dc resistivity (*ρ*_dc_) of the ceramics with various x. (**f**) Resistivity changes with x at 500 °C. (**g**) Relationship between lnρ_dc_ and 1000/T of NBLMTC-x ceramics. (**h**) The activation energy (Ea) of the ceramics is a function of *x* at two temperature regions.

The complex impedance and resistivity of NBLMTC-*x* ceramics were analyzed to understand the conduction mechanisms, as shown in Figure 8a–h. Figure 8a,b depict the impedance spectra for NBLMTC-*x* ceramics with *x* = 0 and *x* = 0.4 across various temperature ranges, respectively. The real part (Z′) and imaginary part (Z″) of the complex impedance are shown. The Cole-Cole semicircle observed in the samples indicates that the grains primarily influence the electrical properties of the piezoelectric ceramics [30,31]. This observation aligns with previous findings regarding the impact of grain size on sample resistance. Besides, the semicircles in the complex impedance plots for NBLMTC-*x* (*x* = 0) and NBLMTC-*x* (*x* = 0.4) shrink with increasing temperature decreasing resistance. Notably, NBLMTC-*x* (*x* = 0.4) exhibits a larger complex impedance, suggesting Ce doping effectively enhances the ceramics’ voltage resistance, improving overall resistance. 

In order to further analyze the electrical properties of NBLMTC-x ceramics, the electrical impedance spectra of the components with different Ce^4+^ doping concentrations were thoroughly analyzed. Figure 8c,d shows the frequency dependence of the imaginary part (Z″) of the complex impedance of NBLMTC-x ceramics tested at frequencies ranging from 100 Hz to 1 MHz at different temperatures. The results show that at high frequencies (>10 kHz), all the impedance curves tend to merge, implying a significant decrease in the impedance properties of the material with increasing temperature due to the release of space charge. Furthermore, for all components, two characteristic peaks show in Z″ at low frequencies (<10 kHz); the double peaks suggest that the contribution of grains and grain boundaries determines the impedance behavior of the samples. Besides, it is abundantly evident from the figure that the maximum value of the imaginary component of the complex impedance (Z″_max_) falls with increasing temperature, together with the broadening of the peaks, implying a temperature-dependent relaxation phenomenon in the dielectric behavior [34]. Z″_max_ moves to the high-frequency side as the temperature rises, which suggests even more that the relaxation time (ω_m_τ_m_ = 1) reduces with rising temperature. The variation of the eigenfrequency corresponding to Z″_max_ (*f*_m_ = 2πω_m_) with temperature satisfies Arrhenius’ law [35,36]:

(4) $$f_{m}=f_{0}exp\left(\frac{-E_{a}}{K_{B}T}\right)$$

where *f_m_* is the eigenfrequency; *f*_0_ is the exponential prefactor; *E_a_* is the activation energy; *K*_B_ is Boltzmann’s constant; *T* is the thermodynamic temperature.

Figure 8e illustrates the DC resistivity of NBLMTC-x ceramics changes with temperature. The resistivity of these ceramics decreases progressively with increasing temperature, with the sample having x = 0.4 exhibiting the highest resistivity of 1.0 × 10^8^ Ω·cm at 500 °C, consistent with the trends observed in the complex impedance data. Figure 8f shows the resistivity of NBLMTC-x ceramics at 500 °C. Ce doping greatly improves the resistivity by one to two orders of magnitude. The observed increase in complex impedance and conductivity can be attributed to two main factors. First, the substitution of (LiMn)^5+^ for Bi ions at the A-site in NBT effectively reduces Bi volatilization, decreases oxygen vacancy concentration and minimizes defects, thereby enhancing the voltage resistance of the samples. Second, Ce doping reduces grain size and increases grain boundary density, extending the carrier conduction path and improving overall resistivity. Figure 8g shows the relationship between lnρ_dc_ and 1000/T of NBLMTC-x ceramics. The temperature range is divided into low-temperature and high-temperature zones, with the boundary at 350 °C. Both lnρdc and 1000/T of the two ceramic samples show a linear relationship, indicating that there is only one conduction mechanism in the temperature range tested. Figure 8h summarizes the variation in *E*a with Ce doping, as determined by the Formula (4). It can be clearly seen that in the low-temperature region, activation energies range from 0.65 to 0.86 eV, while in the high-temperature region, the activation energy ranges from 0.80 to 1.31 eV. This result indicates that at lower temperatures, conductivity is primarily influenced by external semiconductor carriers, whereas at higher temperatures, the conductivity is governed by intrinsic mechanisms [37,38].

**Figure 9:**
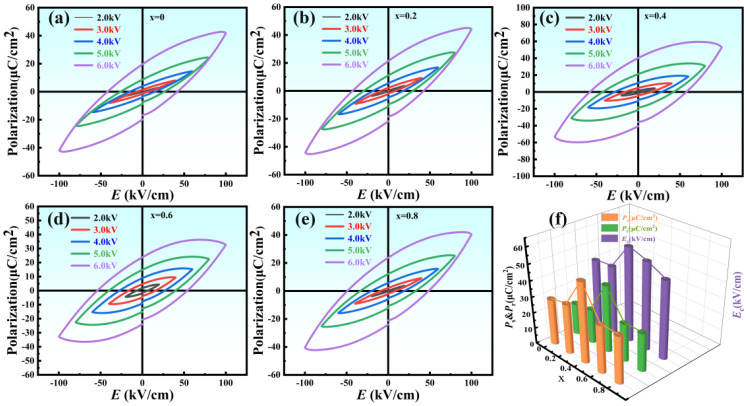
(**a**–**e**) P–E hysteresis loops of NBLMTC-x ceramics under various electric fields. (**f**) Variation of *P_r_*, *E_c_* and *P_s_* with respect to x at 100 kV/cm.

The *P*–*E* hysteresis loops of NBLMTC-x ceramics at various electric fields were measured at 1 Hz and room temperature, as shown in Figure 9a–e. All samples exhibited typical *P*–*E* hysteresis behavior. The figures show that the *P*–*E* loop progressively approaches saturation as the electric field increases and the residual polarisation is enhanced. The loop reaches full saturation and achieves its maximum residual polarisation at ±100 kV/cm electric field strength. This saturation behavior across different NBLMTC-x ceramics indicates that Ce doping effectively enhances the ferroelectric properties of the ceramics. This is because the replacement of Ti^4+^ by Ce^3+^ (or Ce^4+^), where Ce^3+^ (or Ce^4+^) functions as a donor, leads to some vacancies in the lattice, which facilitates the movement of domain wall so as to improve the piezoelectric properties significantly [39]. In addition, remanent polarisation (*P_r_*) exhibits a pattern of increasing and then decreasing variation, which is the same as that of the piezoelectric coefficient with doping x (see Figure 9f). It is shown that the introduction of Ce ions can induce more ferroelectric domain flipping, which is helpful to the flipping of polarization and thus improves the ferroelectric and piezoelectric properties. In general, tetraval distortion and distortion will help to obtain fuller polarization, and fuller polarization will help to reduce the unstable non-180° domains in the ceramic, which will help to improve the thermal stability of the ceramic. The dependence of the remanent polarisation (*P_r_*), coercive field (*E_c_*) and spontaneous polarisation (*P_s_*) on composition at ±100 kV/cm is summarised in Figure 8f. *P_r_* and *E_c_* initially increase with Ce doping, reaching their peak values before subsequently decreasing. Specifically, the sample with *x* = 0.4 exhibits the highest *P_r_* (*P_r_* = 41.7 μC/cm^2^) and coercive field (*E_c_* = 58.6 kV/cm). This indicates that the x = 0.4 samples can endure higher electric field strengths and exhibit superior piezoelectric properties. Consequently, Ce doping enhances the electrical breakdown strength of the NBLMTC-*x* samples, allowing for further polarisation and thereby improving the ceramics’ ferroelectric and piezoelectric properties.

**Figure 10:**
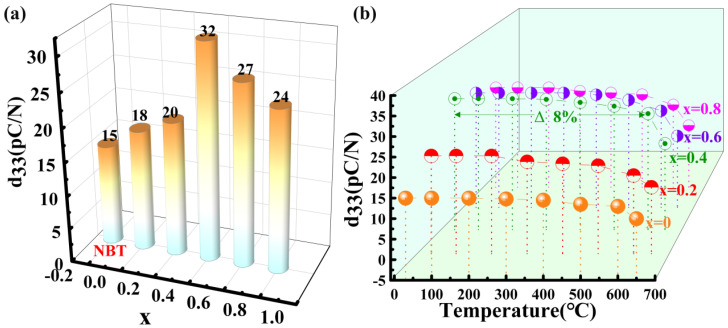
(**a**) Variation of piezoelectric constant (*d*_33_) value with doping *x* at room temperature. (**b**) Variation of *d*_33_ with temperature for NBLMTC-x ceramics.

Figure 10a shows the variation of *d*_33_ with doping level x for NBLMTC-*x* ceramics. The *d*_33_ value initially increases and then decreases with higher Ce doping, increasing from 15 pC/N at *x* = 0.0 to 32 pC/N at *x* = 0.4. This trend indicates that Ce doping substantially enhances the piezoelectric activity of NBT ceramics. The improved piezoelectric performance can be attributed to enhanced ferroelectric properties and better electrical domain alignment. Furthermore, the effect of Ce^4+^ substitution for Ti^4+^ on Ti–O octahedral distortion, grain size and oxygen vacancy concentration collectively influence the structural characteristics and piezoelectric properties of NBLMTC-x ceramics. The effectiveness of piezoelectric materials also depends on their temperature stability, which is crucial for their performance in high-temperature applications. Therefore, the sample was tested in situ for *d*_33_ at 25 °C~650 °C using a variable temperature *d*_33_ tester. As shown in Figure 10b, all samples exhibited varying *d*_33_ temperature dependencies over the measured range. NBLMTC-*x* (*x* = 0.4) ceramics demonstrated the best thermal stability, maintaining high *d*_33_ values at 25 °C~600 °C. In the entire temperature range, the *d*_33_ of NBLMTC-*x* (*x* = 0.4) ceramics drifted by less than 8%. This exceptional thermal stability of NBLMTC-*x* (*x* = 0.4) ceramics makes them well-suited for applications in demanding, high-temperature environments. High resistivity and elevated *T_C_* are essential for piezoelectric ceramics intended for use at high temperatures. Because Ce-doped NBT ceramics simultaneously exhibit high *T_C_*, *d*_33_ and resistivity at 25 °C~600 °C, they are promising for applications in high-temperature piezoelectric devices.

## 4. Conclusions

Na_0.5_Bi_3_(LiMn)_0.9_Ti_4−*x*_Ce*_x_*O_15_ (*x* = 0, 0.2, 0.4, 0.6 and 0.8) ceramics were synthesized via solid-phase processing. Substituting Ce for Ti^4+^ on the B-site and replacing Bi with (LiMn)^5+^ on the A-site effectively improved the piezoelectric constants, resistivity and thermal stability of the NBT-based ceramics. The optimized cerium doped NBT ceramic weakens the orthogonal distortion of the lattice, thereby enhancing the residual polarization of NBLMTC-*x* ceramic, with a piezoelectric constant of up to 32 pC/N and a curie temperature of 648 °C. Additionally, the piezoelectric constant *d*_33_ of Na_0.5_Bi_3_(LiMn)_0.9_Ti_3.6_Ce_0.4_O_15_ demonstrates excellent thermal stability, varying by only 8% in the temperature range of 25 °C~600 °C. Finally, The Ce-doped NBT ceramics effectively reduce the grain size of the ceramics, and the small grain size of these ceramics gives them a resistivity of up to 1.2 × 10^8^ Ω·cm (at 500 °C). The excellent overall performance of the optimized Ce-doped NBT ceramics meets the requirement of sensors’ piezoelectric elements, simultaneous large piezoelectric constants and high resistivity, mend problems facing high-temperature acceleration sensors.

## Data Availability

The raw data supporting the conclusions of this article will be made available by the authors on request.
